# Beneficial Effects of RNS60 in Cardiac Ischemic Injury

**DOI:** 10.3390/cimb44100331

**Published:** 2022-10-14

**Authors:** Magdalena A. Zabielska-Kaczorowska, Barbara Wierzbicka, Andreas Kalmes, Ewa M. Slominska, Magdi H. Yacoub, Ryszard T. Smolenski

**Affiliations:** 1 Department of Biochemistry, Medical University of Gdansk, 80-210 Gdansk, Poland; 2 Department of Physiology, Medical University of Gdansk, 80-210 Gdansk, Poland; 3 Department of Cardiac and Vascular Surgery, Medical University of Gdansk, 80-210 Gdansk, Poland; 4 Revalesio Corporation, Tacoma, WA 98421, USA; 5 Faculty of Medicine, National Heart & Lung Institute, Imperial College London, London SW7 2BX, UK

**Keywords:** RNS60, cardiac ischemic injury, cardiac hypoxia, cardiac energetics, cardioplegia, Custodiol

## Abstract

RNS60 is a physically modified saline solution hypothesized to contain oxygen nanobubbles. It has been reported to reduce ischemia/reperfusion injury in a pig model of acute myocardial infarction. We investigated the effects of RNS60 during cardiac hypoxia in mice and as an additive to cardioplegic solution in rat hearts. ApoE^−/−^LDLr^−/−^ mice were treated by intravenous injection of RNS60 or saline as a control while monitoring the ECG and post-hypoxic serum release of troponin T and creatine kinase activity. Hearts infused with Custodiol containing 10% RNS60 or saline as the control were subjected to 4 h of 4 °C preservation, followed by an assessment of myocardial metabolites, purine release, and mechanical function. RNS60 attenuated changes in the ECG STU area during hypoxia, while the troponin T concentration and creatine kinase activity were significantly higher in the serum of the controls. During reperfusion after 4 h of cold ischemia, the Custodiol/RNS60-treated hearts had about 30% lower LVEDP and better dp/dt_max_ and dp/dt_min_ together with a decreased release of purine catabolites vs. the controls. The myocardial ATP, total adenine nucleotides, and phosphocreatine concentrations were higher in the RNS60-treated hearts. This study indicates that RNS60 enhances cardioprotection in experimental myocardial hypoxia and under conditions of cardioplegic arrest. Improved cardiac energetics are involved in the protective effect, but complete elucidation of the mechanism requires further study.

## 1. Introduction

Effective protection against ischemic injury remains an important challenge in the cardiovascular field. Providing oxygen during such incidents is an obvious strategy, but its accomplishment is difficult. The role of oxygen in myocardial metabolism is complex and can either be beneficial or contribute to cardiac dysfunction and death [1]. With a lack of oxygen, the energy produced by the metabolism of a variety of metabolic fuels, including fatty acids, glucose, lactate, ketones, and amino acids, is insufficient to meet cardiac needs [2]. On the other hand, oxygen can induce irreversible cellular damage and death through the generation of reactive oxygen species (ROS) [3]. It has been shown that oxidative stress is involved in a variety of degenerative processes, syndromes, and diseases where cell transformation or mutagenesis leads to cancer, atherosclerosis, ischemic heart disease, stroke, rheumatoid arthritis, neurodegenerative disorders (Alzheimer’s dementia, Parkinson’s disease), and many more acute inflammatory diseases [4]. Currently, a lot of attention is focused on mitigating oxidation-linked diseases or disorders through antioxidant compounds. RNS60 is not, per se, an anti-oxidant compound. It does not seem to induce oxidative stress but consists of oxygen-containing nanobubbles and supports cellular oxidative energy metabolism [5].

RNS60 is a novel therapeutic agent generated by subjecting 0.9% sodium chloride solution to a process involving Taylor–Couette–Poiseuille (TCP) flow in a rotor/stator device under elevated oxygen pressure. RNS60 is hypothesized to contain oxygen nanobubbles, which have also been referred to as charge-stabilized nanostructures. The gas/liquid interface and energy transfer during the processing of RNS60 are maximized using a rotor equipped with cavities. The specific conditions used are thought to develop a strong shear layer at the gas/liquid interphase and to be associated with the generation of small bubbles from cavitation. RNS60 nanobubbles are stable for extended periods of time when RNS60 is stored in closed containers under refrigeration [5]. As RNS60 contains no pharmacological additives, it may be safely utilized in many pathological conditions, e.g., neuroinflammatory, neurodegenerative, and cardiovascular disorders. In cell cultures and MPTP-intoxicated mice (an animal model of Parkinson’s disease), treatment with RNS60 demonstrated powerful anti-inflammatory and neuroprotective properties [6]. It was shown that RNS60 suppressed neuronal apoptosis, attenuated Tau phosphorylation, and protected memory skills in an animal model of Alzheimer’s Disease [7]. Moreover, RNS60 treatment resulted in an ameliorated adoptive transfer of experimental allergic encephalomyelitis, a model of multiple sclerosis, in vivo [8]. Additionally, it altered Xenopus laevis oocyte biophysical membrane properties by enhancing mitochondrial ATP production [9]. Recent studies have demonstrated that treatment with RNS60 had the potential to inhibit the apoptosis of cardiomyocytes subjected to ischemia/reperfusion (I/R) injury and reduced I/R injury in vivo in pig hearts [10,11]. However, the profile of metabolic changes in the ischemic heart following the infusion of RNS60 has not been characterized. Therefore, we examined the influence of RNS60 in a mouse model of cardiac ischemia. We and other scientific groups have previously demonstrated that Apolipoprotein E and LDLR receptor double knock-out (ApoE^−/−^LDLr^−/−^) mice develop similar symptoms to ischemic injury when exposed to hypoxia challenge [12,13,14]. The other scientific approach was to check the influence of RNS60 in cold ischemia/reperfusion injury in isolated hearts [15]. During cardiac transplantation, the ischemic tolerance is short and ranges from 4 to 6 h. This period of time is important in heart transplantation procedures and acceptance of the transplant [16]. Many efforts have been made to improve preservation solutions and, as a consequence, to extend the limitations of time. The best-known preservation solutions are Custodiol, Celsior, and the University of Wisconsin solution, which contain additions that prevent ischemic cell damage, such as mannitol, glutamate, or histidine but not oxygen [17,18]. The purpose of this work was to determine the potential of RNS60 added to a preservation solution to ameliorate injury due to cold storage and reperfusion. 

Summing up, in this study we evaluated ECG STU area changes, creatine kinase activity, and troponin T release in a mouse model of cardiac hypoxia. Furthermore, we tested RNS60 in the rat model of cardioplegic arrest and its impact on nucleotide catabolite release and the mechanical function of the heart.

## 2. Materials and Methods

All institutional and national guidelines for the care and use of laboratory animals were followed and approved by the appropriate institutional committees (No POIG.01.01.02-00-069/09; 11 July 2011). Mice and rats were housed in 12 h/12 h light/dark cycle in individually ventilated cages (23 °C, 40% humidity) and fed with a standard chow diet. The mice were anesthetized with 100 and 10 mg/kg and the rats with 60 and 10 mg/kg mixtures of ketamine and xylazine given intraperitoneally. RNS60 was donated by Revalesio Corporation (Tacoma, WA, 98421, USA). Male ApoE^−/−^LDLr^−/−^ mice on the C57BL/6J x 129/SvJ background were obtained from Taconic (Ejby, Denmark). For the experiments, one-year-old male mice were assigned to 2 groups. Male Wistar rats (n = 10) weighing 350 g were used in the study. Rat hearts were excised and immersed at a proportion of 9:1 in Custodiol (Dr. Franz Köhler Chemie GmbH, Bensheim, Germany) with 0.9% sodium chloride (n = 5) or Custodiol with RNS60 (n = 5). The Custodiol cardioplegia contained 9 mM KCl, 15 mM NaCl, 4 mM MgCl_2_, 1 mM KH_2_PO_4_, 2 mM tryptophan, 180 mM histidine, 30 mM mannitol, 1 mM ketoglutarate, and 0.015 mM CaCl_2_, with pH 7.02–7.2 at 4 °C. Hearts were perfused in the Langendorff mode with Krebs–Henseleit buffer consisting of 4.8 mM KCl, 118.5 mM NaCl, 1.2 mM MgSO_4_, 1.36 mM KH_2_PO_4_, 1.36 mM CaCl_2_, 25 mM NaHCO_3_, and 11 mM glucose. The buffer was oxygenated at 37 °C by gassing the solution with 95% O2/5% CO_2_.

### 2.1. Cardiac Hypoxia in Mice

The mice were intubated and connected to the respirator. After stabilization, the oxygen supply was reduced from 21% to 8% oxygen and established for 9.5 min. In the 3.5 min of hypoxia, when STU changes in the ECG were clearly visible, the tested compounds were intravenously injected in the slow infusion. One group of mice was injected with a 200 µL bolus dose of RNS60 (n = 5) and another with 200 µL 0.9% sodium chloride (n = 5) as a control group. After the hypoxic episode, the oxygen content was taken back to 21% and the mice were allowed to recover. Before, during, and after the hypoxic stress, the ECG was recorded using the bipolar electrodes of Biopac Systems MP 150 (Goleta, CA 93117, USA) connected to a PC running AcqKnowledge 3.7.2 Biopac software. 

### 2.2. Troponin T Concentration and Creatine Kinase Activity in Mouse Serum after the Hypoxic Event

Peripheral mice blood was obtained 5 h after the drug administration from the lower main vein. The blood was clotted and serum was obtained by centrifugation at 4000 rpm for 10 min at a temperature of 21 °C. The troponin T concentration was detected with a standard immunochemical assay (Sun Red Mouse Troponin T ELISA Kit) according to the manufacturer’s instructions. Creatine kinase (CK) activity was calculated by an enzymatic method [14]. The serum was incubated in a buffer containing (in mmol/L) 300 phosphocreatine, 5 AMP, 10 Mg 2+, 100 imidazole acetate, 2 CEP, and 20 N-acetyl cysteine at pH 6.7 and a temperature of 30 °C. The reaction was terminated by the addition of 1.3 M HClO_4_, followed by 2M KOH neutralization. The creatine and phosphocreatine concentrations were estimated with RP-HPLC [19].

### 2.3. Cardioplegic Arrest of the Rat Heart

After the anesthesia reached a steady state, the rat hearts were excised quickly and placed in ice cold sodium chloride. The cardioplegic arrest of the rat hearts was performed with a Custodiol cardioplegic solution commonly used at the Medical University to preserve organs undergoing transplantation. The hearts were initially perfused for 5 min at a temperature of 4 °C. The hearts were arrested by a perfusion of cold (4 °C) Custodiol cardioplegic solutions with 0.9% sodium chloride (n = 5) and Custodiol with 10% RNS60 (n = 5) at a constant pressure of 60 cm H_2_O for 5 min. Finally, they were immersed in a cardioplegic control solution or cardioplegia with RNS60 and stored for 4 h at 4 °C. At the end of the preservation period, after 4 h, the hearts were perfused with Krebs–Henseleit buffer at a constant perfusion pressure of about 100 cm H_2_O. The same balloon catheter was inserted into the ventricle 15 min after reperfusion. Coronary effluent was collected throughout the first 5 min of reperfusion. The volume of the effluent was measured and well-mixed aliquots of 1 mL were taken for the determination of purine release from the myocardium. After 15 min of reperfusion, the mechanical function was evaluated. At the end of the perfusion protocol, the hearts were freeze-clamped and used for nucleotide concentration measurements. 

### 2.4. The Mechanical Function of the Rat Heart

Mechanical function assessment was performed using a balloon catheter inserted into the left ventricle to determine systolic pressure and end-diastolic pressure–volume relations. The balloon was loaded with water from 0 to 200 µL in 25-µL steps. The left ventricular systolic pressure (LVSP), left ventricular end-diastolic pressure (LVEDP), left ventricular systolic pressure (LVDP), dp/dt maximal (dp/dt_max_), and dp/dt minimal (dp/dt_min_) were recorded at all loadings of the balloon. A Biopac Systems MP 100 connected to a PC running AcqKnowledge 3.7.2 Biopac software was used for data acquisition and calculation. 

### 2.5. Sample Preparation and Metabolic Assays

The coronary effluents of the rat hearts were collected during the first 5 min of reperfusion. An aliquot was analyzed by HPLC as described earlier to evaluate the purine catabolite release. The effluents were directly injected into the HPLC system. Freeze-clamped samples of the rat hearts collected at the end of the experiment were freeze-dried and extracted with 0.4 M HClO_4_ with the use of a glass homogenizer placed in the ice. Centrifugation of the homogenates was performed to remove protein precipitates. The supernatant was neutralized with the use of 2 M KOH. The samples were injected into HPLC after the second centrifugation and analyzed as described earlier.

#### Statistical Analysis

All values are expressed as the mean ± SEM. Statistical comparison was performed using Student’s *t*-test for the two examined groups—RNS60 vs. the control group. GraphPad Prism 6 software (GraphPad Software, San Diego, CA, USA) was used for all statistical tests, and a value of *p* < 0.05 was considered significant.

## 3. Results

### 3.1. Electrocardiographic, Troponin T, and Creatine Kinase Activity Changes during and after Hypoxic Stress in ApoE^−/−^LDLr^−/−^ Mice

**Figure 1:**
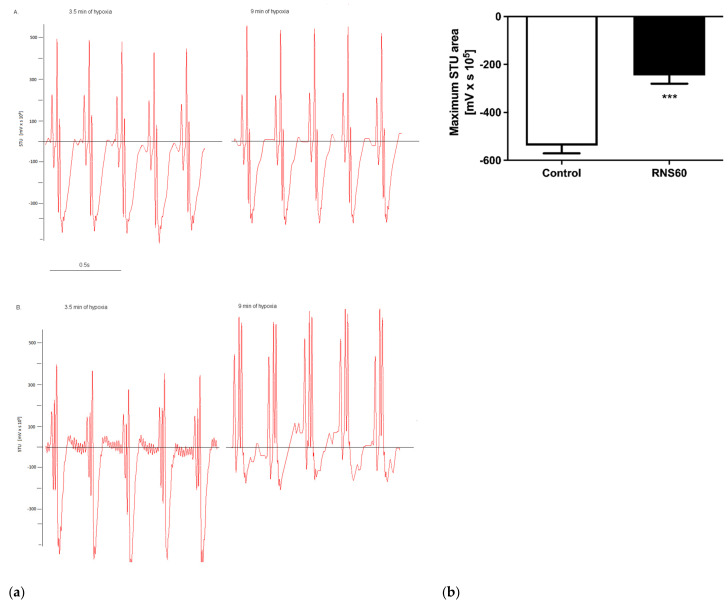
ECG. (**a**) A. At 3.5 min of hypoxic stress. B. At 9 min of hypoxic stress. (**b**) STU area in the 9th min of hypoxic stress in ApoE^−/−^LDLr^−/−^ mice that received the intravenous injection of RNS60 or saline as the control. Values represent mean ± SEM (n = 5); *** *p* < 0.0001.

In the ApoE^−/−^LDLr^−/−^ mice, myocardial ischemia was manifested by typical changes in the ST segment 3.5 min after hypoxia induction. At this time point, normal saline (0.9% sodium chloride) or RNS60 was slowly injected intravenously. Visible differences in the ECG were observed between the RNS60 and the control groups (Figure 1a). The control group displayed significant STU depression at an 8% oxygen concentration, while changes in the RNS60 group were minor. Immediately after the cessation of hypoxia, the ECG was reversed, and 5 min later, the hypoxic event alterations were back to normal. This procedure generated reversible ischemic stress rather than a sustained myocardial infarction in both investigated groups. The ischemic burden was calculated as the STU area. The STU area is the area covered by the STU (repolarization) segment shift from the isoelectric line and was calculated manually (Figure 1b). It was significantly higher in the control group of mice. 

**Figure 2:**
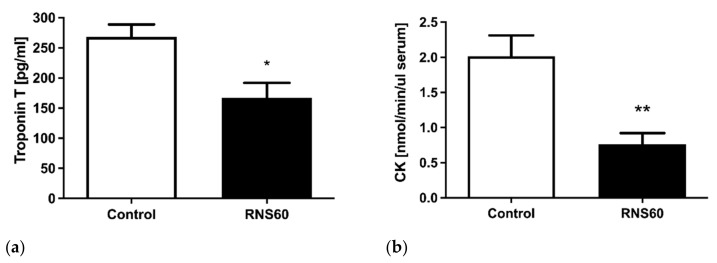
Changes in biomarkers of hypoxia. (**a**) Serum troponin T concentration, (**b**) serum creatine kinase activity in ApoE^−/−^LDLr^−/−^ mice that received intravenous injection of RNS60 or saline as control 5 h after the hypoxic event. Values represent mean ± SEM (n = 5); * *p* < 0.02, ** *p* < 0.006.

Infarction marker analysis in serum revealed significantly (20%) lower troponin T levels after 5 h of hypoxic stress in the RNS60 group as compared to the normal saline-treated group (Figure 2a). Creatine kinase activity decreased in the RNS60 group vs. the normal saline group by 35% (Figure 2b).

### 3.2. The Mechanical Function of the Rat Heart

**Figure 3:**
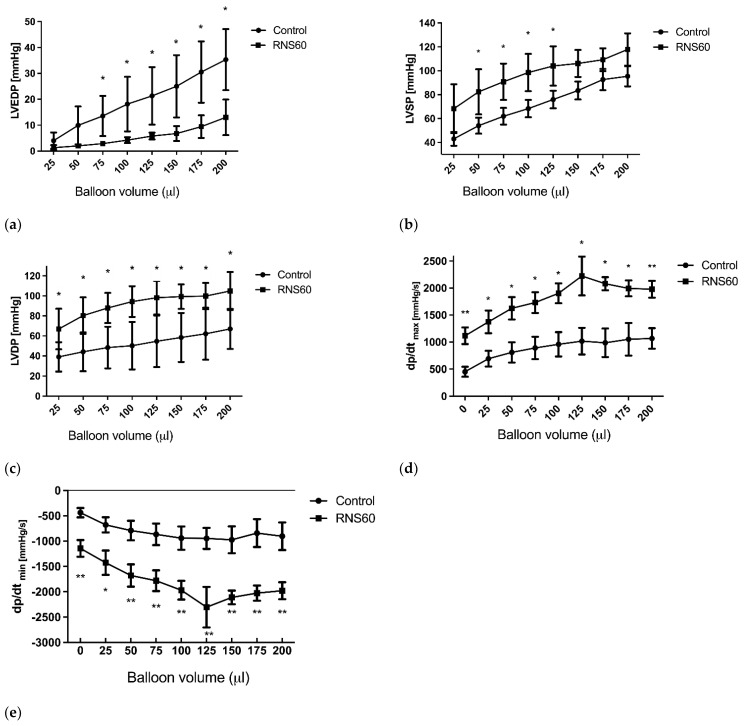
(**a**) Left ventricular end-diastolic pressure (LVEDP), (**b**) left ventricular systolic pressure (LVSP), (**c**) left ventricular systolic pressure (LVDP), (**d**) values of dp/dt_max_, and (**e**) values of dp/dt_min_- volume relation of the rat heart after 4 h of hypothermic ischemia in the control and the group that received cardioplegia with RNS60. Values represent mean ± SEM (n = 5); * *p* < 0.01, ** *p* < 0.05.

During the reperfusion phase, LVEDP, which is related to the stiffness of the ventricle, was decreased in the RNS60 group compared to the normal saline control group at each volume load in the balloon (Figure 3a). Comparison of the recovery of the mechanical function expressed as LVSP or developed pressure showed significant differences between the two examined groups, and it was increased in the RNS60 group compared to the control group (Figure 3b). The LVDP was raised in the all balloon loads in the RNS60 group (Figure 3c). Both the dp/dt max and dp/dtmin were increased in the RNS60 group (Figure 3d,e).

### 3.3. Metabolic Markers of Injury in Rat Heart

**Figure 4:**
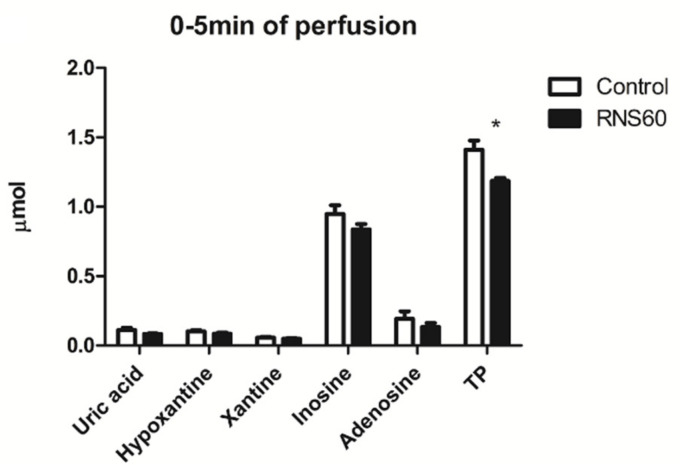
The release of purine catabolites from the rat heart during the initial 5 min of reperfusion after prolonged hypothermic ischemia in the control and group that received cardioplegia with RNS60. Values represent mean ± SEM (n = 5); * *p* < 0.02.

The release of total purine catabolites from the rat hearts during the first 5 min of reperfusion was significantly (26%) lower in the RNS60 group (Figure 4). 

**Figure 5:**
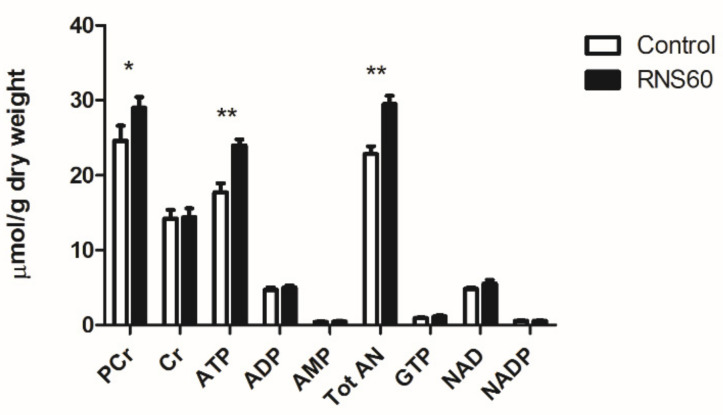
The myocardial concentration of nucleotide and creatine metabolites in the rat heart at the end of protocol involving 4 h of hypothermic (4 °C) ischemia and 15 min of reperfusion in the control and group that received cardioplegia with RNS60. Values represent mean ± SEM (n = 5); * *p* < 0.01, ** *p* < 0.003.

No significant differences in the concentration of other metabolites in the effluents were observed. Myocardial ATP concentration was significantly higher (28%) in the RNS60 group as was the total adenine nucleotide pool (22%) and phosphocreatine (25%) after 4 h of ischemia and reperfusion. There was no significant difference between the hearts concerning the myocardial content of other metabolites (Figure 5). 

## 4. Discussion

This study demonstrates that RNS60 reduced hypoxia-induced myocardial injury and produced a cardioprotective effect in combination with cardioplegic arrest and hypothermia. 

It has been shown earlier in multiple disease models, in preclinical in vitro and in vivo studies, that RNS60 has broad anti-inflammatory and cytoprotective effects [5,6,20,21]. Here, we used ApoE^−/−^LDLr^−/−^ mice known to respond to exposure to hypoxia with the induction of electrocardiographic changes and the elevation of serum troponin T concentrations, indicating acute myocardial infarction [12,22]. Exposure to hypoxia resulted in profound alterations in the control mice treated with saline, while the administration of RNS60 prevented these deleterious changes. These findings indicate that RNS60 had a cardioprotective effect in myocytes that reduced injury resulting from a reduced oxygen supply. This is consistent with previously conducted studies using a balloon-induced myocardial I/R model in pigs [10,11]. The RNS60 treatment caused the normalization of the ECG tracings and reduced mortality in male pigs. Additionally, it was previously found that the combination of troponin T and CK activity measurements had an excellent correlation with the progression of ischemic myocardial injury, including minor myocardial damage [23]. In our experiments, we found lower serum CK activity in the RNS60 group of mice, which is consistent with the troponin T release from the infarcted hearts. To summarize, the treatment with RNS60 has the potential to reduce hypoxic injury in vivo. 

The cardioplegic arrest of the rat heart is the most common system to test different cardioprotective strategies during cardiac surgery and transplantation [24]. There are different approaches to estimating the efficiency of cardioprotective solutions. The first is based on the analysis of coronary effluent collected at different stages of reperfusion [25,26]. Energy conversion of the heart is associated with the dephosphorylation of ATP to ADP, which can be further degraded to AMP and adenosine or, alternatively, to inosine monophosphate and inosine [27]. Cardiomyocytes release nucleosides into the interstitial space, which can be taken up via membrane transport mechanisms by the endothelial cells [28,29]. In the endothelium, nucleosides can be progressively metabolized to inosine, hypoxanthine, xanthine, and uric acid [30]. This study demonstrated that RNS60 decreased the release of purine catabolites during the initial phase of reperfusion. Purine content was almost 20% lower in the coronary effluent collected during the first 5 min of reperfusion after prolonged hypothermic ischemia. Purine catabolite release in perfusion systems is a very sensitive measure of ischemic damage. It correlates closely with the extent of adenine nucleotide pool depletion in the heart. It was previously found by Ogino and Katayama that a rapid washout of purine precursors at reperfusion did not provide substrates with nucleotide synthesis in the heart. This is closely linked to difficulties with the restoration of contractile function [25,31].

The second approach to estimating the efficiency of cardioplegic solutions is the measurement of the myocardial energy-rich phosphates in the heart homogenates. For instance, the ATP concentration directly correlates with the activity of the contractile apparatus, and the depressed level of ATP is used as a prognostic factor in unsettled functional recovery of the heart [32,33,34]. Severe nucleotide depletion was linked with poor recovery, but on the other hand, mild depletion was not important in the case of functional impairment [35]. It was previously shown that RNS60 positively modulated synaptic transmission by up-regulating ATP synthesis, leading to synaptic transmission enhancement at the squid giant synapse in Xenopus laevis oocytes [36]. Additionally, RNS60 altered biophysical membrane properties by enhancing mitochondrial ATP production in Xenopus laevis oocytes [9]. In our study, both the ATP concentration and ATP/ADP ratio in the heart were significantly higher in the RSN60 group at the end of reperfusion in comparison to the control group. It was previously found that in failing hearts, the ATP concentration was 30% lower than in healthy organs [34]. This is in contrast to the findings about RNS60 treatment, which had a positive influence on the ATP content. It was found in canine and human experiments that the oxygenation of a crystalloid cardioplegic solution enhanced myocardial recovery and protection during ischemic cardioplegic arrest [35]. Post-reperfusion ATP levels were 62% and 89% of the baseline in hearts that received an aerated crystalloid solution in comparison to hearts that were treated identically, except that the crystalloid cardioplegic solution was fully oxygenated. Furthermore, in our studies, phosphocreatine concentration was also higher in the RNS60-treated group. These findings indicate that the energetics were improved in the hearts perfused with RNS60. Previous experiments have found a lower creatine pool in failing human myocardium [36]. Moreover, a decrease in total creatine was observed in the hearts with dilated and hypertrophic cardiomyopathy [37,38]. In our study, all hearts recovered mechanical function after 4 h of ischemia. Consistent with the improved energy status of the heart, both systolic (dP/dt_max_, LVDP, LVSP) and diastolic functions (dP/dt_min_, LVEDP) were better in the hearts infused with the RNS60 solution. Physiological consequences included an increased capacity for mechanical work and contractile reserve, rendering the RNS60 hearts more resistant to ischemic injury.

## 5. Conclusions

In conclusion, our experiments indicate that RNS60 protected mouse hearts from hypoxia-induced damage by decreasing creatine kinase and troponin T release. Moreover, as an adjunct to cardioplegic fluid, RNS60 can extend the protective effect of the procedure. Our study demonstrates the critical role of RNS60 in cardiac nucleotide metabolism under ischemic conditions. RNS60 improved the cardiac metabolic equilibrium of the heart mainly through the protection of the ATP pool and lower purine release. Moreover, the improvement in both systolic and diastolic post-ischemic cardiac function was observed in the RNS60 rat hearts. As we discovered, the early reperfusion period cannot be immediately extrapolated to clinical conditions, but the differences may be of clinical relevance, especially in the case of cold-storage times greater than the usual 4 h. Overall, RNS60 could be a therapeutic agent to preserve cardiac energy homeostasis and improved cardiac function in patients with heart disease.

## Data Availability

Not applicable.
